# Editorial: Experimental Approaches to Body Image, Representation and Perception

**DOI:** 10.3389/fpsyg.2021.809385

**Published:** 2021-12-07

**Authors:** Kevin R. Brooks, Jason Bell, Lynda G. Boothroyd, Ian D. Stephen

**Affiliations:** ^1^Body Image and Ingestion Group, Faculty of Medicine, Health and Human Sciences, Macquarie University, Sydney, NSW, Australia; ^2^School of Psychological Sciences, Faculty of Medicine, Health and Human Sciences, Macquarie University, Sydney, NSW, Australia; ^3^Perception in Action Research Centre, Faculty of Medicine, Health and Human Sciences, Macquarie University, Sydney, NSW, Australia; ^4^School of Psychological Science, University of Western Australia, Perth, WA, Australia; ^5^Department of Psychology, Durham University, Durham, United Kingdom; ^6^School of Social Sciences, Nottingham Trent University, Nottingham, United Kingdom

**Keywords:** body image (MeSH), perception, body representation, experimental, clinical psychology

The study of mental representations of bodies stretches back to at least 1905 when French neurologist Pierre Bonnier used the word *aschematie* to refer to a loss of awareness of parts of the body (Bonnier, 1905/2009). Credit is often also given to another neurologist—Englishman Sir Henry Head—whose definition of body schema remains influential to this day (Head et al., 1920). While early studies such as these concentrated on anomalies of body perception and experience following brain injury or amputation, the importance of a multidisciplinary approach was soon realized. Although the classic work of Schilder may be best remembered for its enduring definition of body image (“the picture of our own body which we form in our mind…”), it is also significant for recognizing the importance of sociocultural and psychological considerations alongside the dominant neurological approach of the time (Schilder, 1935/1950). Since then, a broad variety of academics have endeavored to define, measure, and understand this complex and multidimensional construct. The diversity of studies in the current Research Topic reflects this interdisciplinary approach. First, this is demonstrated by the participating journals and sections. Four journals and seven unique sections were involved: Frontiers in Psychology (sections devoted to Cognition, Health Psychology, Cognitive Science, and Psychology for Clinical Settings); Frontiers in Nutrition (Eating Behavior); Frontiers in Neuroscience (Perception Science), and Frontiers in Psychiatry (Psychopathology). The nationality of the groups of authors demonstrates further diversity. In all, 83 contributing authors were based in 15 different nations across five continents. The breadth of participant groups, methods, and topics further demonstrate the multifaceted nature of contemporary research on body image, representation, and perception.

The articles in this Research Topic can be grouped into several overlapping themes. A number of articles are concerned with establishing effective and rigorous systems of measurement for the various aspects of behavior connected with body image disturbance. Korn et al. employ the conjoint analysis (CA) method to measure fear of weight gain, while Legenbauer et al. introduce the Body Image Approach Test (BIAT): a behavioral assessment task for Body Avoidance and Body Checking. Meanwhile, Alexi et al. describe an assessment of the use of synthetic (computer-generated) body stimuli in measuring body size estimation, replicating the serial dependence effect that they previously demonstrated with photos of human bodies. D'Amour and Harris present a novel method of measuring body size estimation at various viewpoint angles using a psychophysical staircase procedure. In particular, their technique purports to measure the brain's representation of the participant's own body.

The issue of perception and representation is the focus of the Research Topic's only Opinion article (Brooks et al.). This article observes the paradox that the same terminology (body size overestimation) is often used to describe opposite patterns of results by scientists with clinical vs. perceptual psychology backgrounds (particularly those perceptual psychologists that use the adaptation paradigm). Consideration of the assumptions of these sub-disciplines and the type of representations that they believe to be distorted can explain this paradox. The adaptation paradigm, which exposes participants to extreme bodies to cause a bias in size and shape judgements for subsequently seen bodies, is applied in several papers. Ambroziak et al. present a study investigating the locus of the adaptation effect, i.e., whether it affects the perception of the experimental stimuli or the stored representation with which these are compared. Brooks et al. and Gould-Fensom et al. employ this paradigm in investigating the neural representation of body adiposity for different genders or ethnicities, respectively. Brooks et al. demonstrated that body size aftereffects do not transfer completely between male and female bodies, suggesting that the neural populations responsible for body size estimation are somewhat selective for gender. However, Gould-Fensom et al. showed complete transfer of the effect between the bodies of Australians of European descent and Malaysians of Asian descent, suggesting that these neurons are not selective for ethnicity. In addition, this paper reminds us of the importance of including non-WEIRD populations in research on body image and chimes with recent evidence that adaptation effects themselves are not culturally or racially bounded, with ethnically diverse samples in low-media contexts still showing typical aftereffects from viewing high or low weight bodies (Boothroyd et al., 2020). In the same spirit, Thornborrow et al. present a cross-cultural study on body image in relation to muscularity for men in the UK, Uganda, and Nicaragua. Despite similarities in the social pressures to attain such bodies, including exposure to muscular bodies in the media, this study demonstrates differences in the bodies considered to be ideal in these cultures. The majority of research on body ideals concentrates on adiposity, usually using women as participants and female bodies as stimuli. Although muscularity is subject to the same kinds of adaptation effects as adiposity (e.g., Sturman et al., 2017; Brooks et al., 2020; Jacques et al., 2021), these results are a reminder that the nuances of the “ideal” may vary in different ways and may be focused on different body composition dimensions for men and women (Brierley et al., 2016).

Exposure is also a feature of the approach taken by Verfaillie and Daems, who demonstrate that long-term priming confers a reaction time advantage for the discrimination of anatomically possible vs. impossible postures/poses. This advantage generalizes across identity but is dependent on viewpoint angle. D'Argenio et al. also look at the perception of posture, finding that dynamic poses give rise to a perception of increased masculinity/decreased femininity. Ding et al. also look at posture and the event-related potentials caused by bodily expressions of emotions, particularly fear, concluding that emotional changes are processed around 210–260 ms after stimulus onset.

The perception of body posture is tested further by Axelsson et al., who ask whether the superior performance previously demonstrated for upright (compared to inverted) bodies is influenced by the presence of faces when visible. This “inversion effect” is also shown by Nazareth et al., for brief (17 ms) stimuli, when asked to identify human (vs. non-human) body stimuli, and is thought to indicate holistic processing for upright, but not for inverted stimuli. Although, Axelson et al. demonstrate body inversion effects even when faces are not visible, these effects increase when faces are present, suggesting a significant influence of faces on holistic processing for bodies. Ritter et al. also investigate the perception of faces, specifically the presence of an inversion effect for those diagnosed with Body Dysmorphic Disorder (BDD) compared with healthy controls. Although the BDD group were hypothesized to be less holistic in their processing of face stimuli, suggesting a reduced inversion effect, these participants showed no such abnormalities. Face perception is again central to Shi et al., who highlight the facial appearance dissatisfaction of those scheduled to undergo orthognathic (jaw) surgery to address a visible difference.

The link between attention and either the perceptual or attitudinal aspects of body image has been a topic of broad interest in recent years (Cho and Lee, 2013; Lang et al., 2014; Moussally et al., 2016; Stephen et al., 2016, 2018, 2019; Dondzilo et al., 2017, 2018; Berrisford-Thompson et al., 2021). This interest is represented in the current issue by several investigations. Cass et al. use a visual search task to investigate attention as a function of observer body size, demonstrating biases toward bodies matching the observer's own in terms of BMI. Meanwhile, Kim et al.'s approach involves eye movement recording to establish the effects of observing thin-ideal images on restrained eaters' attention to pictures of food. In particular, restrained eaters who scored highly on neuroticism showed increased vigilance for food. Engel et al. use a dot probe task to investigate the possibility that attentional training may redirect participants' tendencies to focus on positive or negative parts of their own bodies. As attentional biases to positive/negative body parts were not clear at baseline, abolition of those biases was not possible. However, it remains to be seen whether an intervention such as this might be effective amongst those with more significant body image concerns (e.g., those diagnosed with eating disorders).

Interventions to improve the attitudinal aspects of body image are the subject of several additional articles in this Research Topic. Kosinski reports a pilot study of an evaluative conditioning app for mobile phones. While healthy subjects showed a promising decrease in body dissatisfaction and drive for thinness alongside an increase in self-esteem, this was no greater in the evaluative conditioning group than in the control condition. In contrast, a dissonance-based eating disorder intervention changed implicit attitudes toward thinness—at least for some participants—in the study by Kant et al. Just as Thornborrow et al. above found differences between populations in how bodies were idealized, this study emphasizes the importance of considering subgroups within populations; there were differences between heterosexual and non-heterosexual women in terms of both baseline scores and the effects of the intervention. To date, the vast majority of work in interventions around body perception and body image has concentrated on younger (typically heterosexual) women in urban and high-income populations. In that context, Sánchez-Cabrero et al. make an important step in further broadening the diversity of the literature by demonstrating the effectiveness of a body dissatisfaction intervention—the IMAGINA program—for a frequently overlooked group: older people. For all the elegance and rigor of basic science investigations, of which there are many in this collection, these translational studies are a reminder of the eventual goal of increasing our understanding of the underlying mechanisms of body image—to make people's lives better. Furthermore, that mission must include the full range of those affected by the appearance pressures to which our distorted perceptual experience gives rise.

As Cash and Smolak (2011) commented: “body image transcends a singular experience. It is complex and multidimensional. It is gendered. It is ethnic and cultural. It is age dependent.” Encompassing both perceptual and attitudinal aspects of body image, the experimental studies in this Research Topic bear out these observations and demonstrate still more aspects of this fascinating construct, demonstrating considerable potential for further growth. It is hard to dispute that over the last century, the study of body image has seen extraordinary development both in breadth and depth, compared to its humble neurological beginnings. Further expansion over the next century seems inevitable, and with this in mind we invite you to submit your research for our next Frontiers Research Topic on body image, currently planned for 2121.

## Author Contributions

KB wrote the original draft. LB, JB, and IS contributed important written material, edited the manuscript, and approved the final draft. All authors contributed to the article and approved the submitted version.

## Conflict of Interest

The authors declare that the research was conducted in the absence of any commercial or financial relationships that could be construed as a potential conflict of interest.

## Publisher's Note

All claims expressed in this article are solely those of the authors and do not necessarily represent those of their affiliated organizations, or those of the publisher, the editors and the reviewers. Any product that may be evaluated in this article, or claim that may be made by its manufacturer, is not guaranteed or endorsed by the publisher.
